# Clinical outcomes of a large-scale, partnership-based regional food prescription program: results of a quasi-experimental study

**DOI:** 10.1186/s13104-023-06280-8

**Published:** 2023-02-10

**Authors:** Nalini Ranjit, Jennifer N. Aiyer, Jack D. Toups, Esther Liew, Kenia Way, Henry Shelton Brown, John Wesley McWhorter, Shreela V. Sharma

**Affiliations:** 1 University of Texas Health Science Center at Houston (UTHealth) School of Public Health, Michael & Susan Dell Center for Healthy Living, Austin, TX USA; 2 University of Texas Health Science Center at Houston (UTHealth) School of Public Health, Houston, TX USA; 3 McGovern Medical School, Houston, TX USA; 4 Save the Children US, Houston, TX USA; 5 Houston Food Bank, Houston, TX USA; 6 Suvida Health Care, Houston, TX USA

**Keywords:** Food prescription programs, HbA1c, Food banks, Food insecurity

## Abstract

**Background:**

Food prescription programs are gaining interest from funders, policy makers, and healthcare payers as a way to provide value-based care. A small body of research suggests that such programs effectively impact health outcomes; however, the quality of existing studies is variable, and most studies use small samples. This study attempts to address these gaps by utilizing a quasi-experimental design with non-equivalent controls, to evaluate clinical outcomes among participants enrolled in a food prescription program implemented at scale.

**Methods:**

We completed a secondary analysis of participant enrollment and utilization data collected between May 2018 and March 2021, by the Houston Food Bank as part of its multi-institution food prescription program. Enrollment data was obtained from 16 health care partners and redemption data from across 40 food pantries in Houston, Texas. Our objective was to assess if program participation impacted multiple cardio-metabolic markers. Exposure was defined as any visit to a food pantry after receipt of prescription. Linear and logistic regression models were used to estimate change in outcomes by exposure status and number of food pantry visits.

**Results:**

Exposed patients experienced a −0.28% (p = 0.007) greater change in HbA1c than unexposed patients, over six months. Differences across exposure categories were seen with systolic blood pressure (−3.2, p < 0.001) and diastolic blood pressure (−2.5, p = 0.028), over four months. The odds of any decline in HbA1c (OR = 1.06 per visit, p < 0.001) and clinically meaningful decline in HbA1c (OR = 1.04 per visit, p = 0.007) showed a linear association with visit frequency.

**Conclusions:**

Our study of a large food prescription program involving multiple health care and food pantry sites provides robust evidence of a modest decline in HbA1c levels among participants. These results confirm that food prescription programs can continue to be effective at scale, and portend well for institutionalization of such programs.

## Background

Poor diet, characterized by the substitution of nutrient-dense fruit, vegetables and whole grains with low cost ‘junk foods’ that are usually calorie-dense and nutrient-poor, is a prominent characteristic of the experience of food insecurity in the United States [1–4]. Chronically poor diet quality, along with cycles of food deprivation and overeating as a result of uncertain availability of food [5, 6], has been pinpointed as a key mediator explaining the higher prevalence of obesity and related diseases, including diabetes, hypertension and cardiovascular disease among food insecure populations [7–9]. Food prescription (Food Rx) programs have emerged as a credible strategy to reduce health disparities, improve healthy food consumption, and reduce disease risk among food insecure populations, particularly in the United States. Unlike most other community food access interventions targeting low income people, these programs have an explicit focus on health conditions, and generally require purposeful participation of healthcare organizations and services [10, 11]. Recommendations for a healthy diet that are primarily initiated within the healthcare context may be more effective at motivating healthy food consumption than those that emanate primarily from food delivery organizations, owing to greater specificity and perceived legitimacy.

A number of studies have attempted to quantify the effectiveness of Food Rx programs in improving diet, measures of chronic disease risk and food security status among participants. However, the quality of these studies is moderate to weak; the majority of these studies are small, conducted in relatively limited settings, and often lack control groups [11–15]. In a recent meta-analysis [16], nine of the 13 included studies did not have a control group, and only one study had a sample size > 150. Although investigators found clinically significant decreases in body mass index (BMI) and glycosylated hemoglobin (HbA1c) (0.6 kg/m2 and 0.8% respectively), the certainty of evidence for these outcomes was graded low to very low [16]. The non-inclusion of comparison groups in most studies is a serious deficit in the extant literature [16]. Pre-post estimates of improvements in health outcomes of patients in a healthcare setting likely overestimate effects of food prescription programs, as these effect estimates include the impacts of other medical interventions targeting those outcomes [16]. The small size of the studies and limited external validity is also a concern, as the attraction of food prescription programs lies partly in their potential for scalability and widespread dissemination [16].

In this study, we present data from a large Food Rx program, implemented by the Houston Food Bank (HFB) and affiliated food pantries in partnership with several healthcare partners in the Greater Houston region. The HFB is the largest food bank in the US, serving over 800,000 individuals each year through 1500 + partnerships across 18 counties in southeast Texas. HFB’s Food Rx program, part of their larger Food for Change program, is designed to deliver healthy foods to improve health outcomes among the historically marginalized members of the community. Our objective in reporting on this program is to provide estimates of effect size that are attainable on cardiometabolic outcomes in a Food Rx program that is implemented at scale.

## Methods

### Study design

This study uses data collected by the Houston Food Bank (HFB) from healthcare organizations and HFB-affiliated food pantries between May 2018 and March 2021. A quasi-experimental approach was employed, wherein Food Rx patients who enrolled and participated in the program were assigned post hoc to the intervention condition, while those who were enrolled but did not participate in the program were assigned to the control group. We conducted secondary data analysis to assess the impact of the HFB Food Prescription intervention (Food Rx) on selected markers of cardio-metabolic health. Data was analyzed by investigators at University of Texas Health Science Center (UTHealth) Houston, which has a data sharing agreement with HFB. The study was approved by the UTHealth Committee for Protection of Human Subjects Institutional Review Board. All methods were performed in accordance with the relevant guidelines and regulations.

### Program description

The HFB has been implementing Food Rx in the Greater Houston area of Texas in partnership with 21 healthcare organizations ranging from small clinics to large healthcare centers. Across all healthcare centers, eligible participants (patients who are food insecure and diagnosed with prediabetes/diabetes, hypertension, or obesity) are invited to enroll in Food Rx, and upon providing informed consent, are provided a unique ID number along with a prescription. The prescription consists of a bi-monthly redemption of ~30 lbs. of fresh produce, plus four ‘Food Rx’ friendly items consisting of whole grains, lean protein and low-fat dairy, as available, to be redeemed at any one of 15 participating HFB food pantries. Participants have leeway in the items they can select within this set of choices. This ‘client choice’ model distinguishes the HFB model from food prescription programs that deliver pre-packaged meals or produce boxes to clients. Food Rx is eligible for redemption twice a month at a food pantry for the duration of time that the participant is enrolled in a wellness program or seeing their provider for medical appointments. Upon arrival at the food pantry for redemption, the participants receive guidance from pantry staff regarding food selection, and indirect nutrition education through nudges, labeling and messaging around the pantry in English and Spanish.

### Data and measures

#### Outcomes data

Since 2019, healthcare partners have been providing HFB with de-identified data on one or more clinical outcomes (primarily HbA1c, but also in some cases, BMI, systolic and diastolic blood pressure (SBP and DBP), and low density lipoprotein (LDL) for patients enrolled into Food Rx, both at the time of recruitment and at follow up visits, where available. Several healthcare partners provide data on only a subset of outcomes, and follow-up outcomes data is frequently not available as patients fail to return to the healthcare partners for follow-up visits. For the analyses presented here, we only included patients who had at least two outcome measurement occasions (complete cases). Within this sample of complete cases, only the first and second outcomes measurements from patients were considered (as pre- and post-data, respectively). The length of time between the first and second measurement varied by patient and healthcare provider, and averaged six months.

#### Exposure data

As all patients presenting at the healthcare partners and meeting the eligibility criteria were enrolled into the Food Rx program, there is as such no planned control group. However, a large subset of patients (47%) that were enrolled by the healthcare partners in Food Rx and provided outcomes data did not redeem their prescriptions at any food pantry during the period between enrollment and first follow up clinic visit. These patients are thus ‘unexposed’ to the Food Rx intervention and can potentially serve as controls to patients who did redeem prescriptions. We also examined alternative measures of exposure, based on different categories of pantry visit frequency, number of redemptions, and intensity of redemptions (visits per month).

#### Demographic data

At the time of enrollment, participants were invited to fill out a survey with demographic data. However, only a subset of healthcare partners made this survey available to patients, and not all patients filled out the survey. As a result, only about a quarter of our analysis sample (432) had demographic data available. Demographic measures obtained included age, gender, education, employment, race/ethnicity, and food security, assessed using the Hunger Vital Sign two-item food insecurity screener, which has been well-validated for use in health care settings [17]. In the absence of data on non-responders, and given the fact that non-completion of the survey depends partly on whether the clinics make the survey available, we assume that participants that completed surveys are not different from participants that did not fill out surveys. Hence, while the demographic measures cannot be included in regression models, they are used here to describe the population.

### Statistical analysis

Sociodemographic characteristics of the surveyed subset (n = 432) of healthcare partners enrollees were examined by (subsequent) exposure status to assess group differences (Table 1). Table 2 estimates are obtained from the analysis sample (i.e., complete cases) for each outcome. Baseline levels and available sample sizes for each outcome, as well as details relating to food pantry visits, are presented separately for exposed and unexposed participants. Mixed-effects linear regression models were used to estimate the change in outcome level across measurement occasions as a function of exposure to food pantries; each of these models included healthcare clinic as a random effect to account for possible clustering of outcomes at the healthcare clinic level and the number of days between measurement occasions as a covariate. Models did not adjust for baseline level of outcome as this was not associated with use of food pantries for any outcome. Mixed-effects logistic regression models explored the odds of any amount of decline, and the odds of clinically significant decline (defined as > 0.50 percentage points [18]), as a function of exposure, for each outcome. Standard errors for all model parameters were based on robust estimators to correct for heteroscedasticity. Supplementary analyses include variations in specification of the outcome variable(s), and are limited to outcomes that show some evidence of an effect of redemption. The threshold for reporting results as significant was a *p* value of 0.05. All analyses were carried out using Stata 17.0 (StataCorp, College Station, TX).

**Table 1:** Demographics of Participants Enrolled in the Food Rx Program, by whether or not they redeemed prescriptions

	Total	0 redemptions	1 + redemptions	p-Value
Sample size	432	250 (58%)	182 (42%)	
Age (mean, SD)	57.1 (14.0)	56.6	57.9	0.365
Size of household (mean, SD)	3.2 (1.7)	3.1	3.2	0.676
Gender				
Female	298 (69%)	167 (67%)	131 (72%)	
Male	134 (31%)	83 (33%)	51 (28%)	0.251
Race/Ethnicity				
Hispanic	241 (56%)	138 (55%)	103 (57%)	
Black	138 (32%)	78 (31%)	60 (33%)	
White, other	53 (12%)	34 (14%)	19 (10%)	0.607
Marital Status				
Married	167 (39%)	88 (35%)	79 (43%)	
Single, divorced, widowed, separated	265 (61%)	162 (65%)	103 (57%)	0.084
Language				
English	266 (59%)	148 (56%)	118 (61%)	
Spanish or other	188 (41%)	114 (44%)	74 (39%)	0.288
Education				
< HS Diploma	186 (43%)	119 (48%)	67 (37%)	
HS Diploma/GED	114 (26%)	63 (25%)	51 (28%)	
> HS Diploma	87 (20%)	44 (18%)	20 (11%)	0.147
Employment Type				
Full Time	76 (18%)	49 (20%)	27 (15%)	
Part Time	56 (13%)	30 (12%)	26 (15%)	
Homemaker/unemployed/retired/other	292 (69%)	167 (68%)	125 (70%)	0.399
Food Insecurity				
Food Secure	64 (17%)	39 (16%)	33 (18.5%)	
Food Insecure	310 (83%)	207 (84%)	145 (81.5%)	0.528
Assistance				
% utilizing SNAP benefits	97 (23%)	58 (23%)	39 (21.4%)	0.663
Fruit/Vegetable Consumption (servings/day) (mean, SD)	0.63 (0.80)	0.59 (0.85)	0.69 (0.77)	0.288

**Table 2:** Details of analysis sample, by clinical outcome

	HbA1c	BMI	LDL	Systolic blood pressure	Diastolic blood pressure
n of patients available for analysis	746	857	224	508	507
n (%) in exposed group	389 (52%)	342 (40%)	112 (50%)	213 (42%)	213 (42%)
Average number of visits among exposed	5.7	4.9	8.9	5.9	5.9
Visits per month among exposed	0.93 (0.8)	1.07 (1.5)	1.14 (1.6)	1.47 (1.9)	1.47 (1.9)
Baseline mean of outcome (full sample)	8.6 (0.3)	39.9 (5.4)	88.3 (1.0)	134.5 (5.6)	79.2 (2.5)
Difference across exposed and control groups	− 0.17 (0.12)	0.14 (0.37)	-1.3 (1.1)	2.4 (1.6)	0.79 (1.6)
p for difference	0.149	0.711	0.79	0.137	0.5
Odds of visiting a food pantry for a unit increase in outcome level (OR, 95% confidence interval)	0.960 (0.911–1.005)	1.001 (0.993–1.009)	0.99 (0.999–0.999)	1.005 (0.998–1.012)	1.007 (0.985–1.030)
p for odds	0.08	0.79	0.79	0.15	0.51
β for number of visits per unit increase in outcome level	− 0.08	− 0.018	− 0.009	− 0.004	− 0.02
p for change in number of visits	0.312	0.1	0.252	0.357	0.337
Average length of pre-post period (full sample)	172	126	202	115	117
Difference across exposed and control groups	19.90	8.89	-0.74	54.86	54.90
p for difference	0.453	0.549	0.888	0.00	0.00

Exposed refers to patients that visited a pantry to redeem their prescription at least once between their first and second clinical measurements; the control group is limited to patients who did not redeem their food prescription at any time between the first and second visits

*HbA1c* glycosylated hemoglobin, *BMI* body mass index, *LDL* low density lipoprotein

## Results

In all, 16 of 21 (76%) healthcare partners provided at least one biometric measure for 2,028 patients during the period between May 2018 and March 2021. About half of the enrolled patients (n = 956, 47%) did not visit any food pantry during the period for which data were available. Patients who did visit a food pantry averaged seven visits, but half these patients did so three or fewer times.

Sociodemographic details of a random sample of enrolled Food Rx patients were obtained via a demographics survey (n = 432) administered at the time of enrollment by the healthcare partners; of these, 42% were subsequently ‘exposed’ (i.e., visited a pantry)^1^ (Table 1). The average age of enrolled patients was 57 years. Nearly 70% of the patients were female. Two in five of the patients had less than high school education, and only about 31% had any type of employment. The population was largely composed of patients of color (56% Hispanic, 32% African-American), and over 80% of patients reported food insecurity at baseline. The sociodemographic composition is largely comparable across those that visited a pantry and those that did not.

Table 2 presents details of the analysis sample (i.e., enrolled patients with at least two different measurement occasions for the given outcome) for each of the five clinical outcome measures, across exposure groups and within the exposed group, where relevant. The largest available sample sizes are for the BMI and HbA1c measures (n = 857 and n = 746 respectively). Across outcomes, the proportion of exposed patients ranges from 40 to 52%, and the number of redemptions varies from 0 to 28. Among exposed patients, the distribution of redemptions is positively skewed. Eighty five percent of participants visited a pantry 12 or fewer times between clinic visits, and 10% visited a pantry more than 18 times (data not shown). On average, exposed patients visited a pantry just once a month between their pre- and post-clinic visits; those with blood pressure measures visited a pantry 1.5 times a month. Thus, the average intensity of exposure among the exposed was approximately at half the prescribed level of two visits each month. Baseline mean of outcome does not differ across exposure groups, suggesting that pantry usage is not selected by severity of baseline measure. The number of visits to a food pantry (i.e., a measure for intensity of exposure) does not differ by outcome level. The average number of months between outcome measurement occasions varies from approximately four months for the systolic and diastolic outcomes, to about six months for HbA1c and LDL outcomes. Except for the two blood pressure measures, the length of the pre-post period does not differ by exposure group. Overall, there is little in these data to suggest that Food Rx enrollees that redeemed prescriptions are clinically different from those that did not visit a pantry.

Table 3 examines if observed change in level of each clinical outcome is associated with any visit to a pantry (primary exposure), number of visits to a pantry (count), and frequency of visits to a pantry (times/month). If exposure is defined as at least one visit to a pantry (with control as no visits), exposed patients experience a −0.28 percent point (*p* = 0.007) greater change in HbA1c than unexposed patients (−0.24 percent point change in unexposed compared to −0.52 percent point change in exposed), over a period of approximately six months. Significant but small differences across exposure categories were also seen with SBP(−3.2, *p* < 0.001) and DBP(−2.5, *p* = 0.028), over a four month period. Pre-post changes in BMI and LDL did not differ by exposure status. A significant linear effect of number of visits on the magnitude of pre-post change was also evident for HbA1c and the two blood pressure measures. The results for the intensity measure (number of visits per month) show that every additional visit per month is associated with significant improvements in levels of HbA1c, systolic and diastolic blood pressure, and significant negative impacts on LDL levels. While systolic and diastolic blood pressure measures showed some improvements, the observed changes were likely too small to be clinically meaningful at any level of pantry usage.

**Table 3:** Associations between measures of food pantry usage and changes in clinical outcomes

	HbA1c	BMI	LDL	Systolic blood pressure	Diastolic blood pressure
Pre-post change in outcome level					
Control group (did not visit a pantry)	− 0.24 (0.12)	0.25 (0.23)	− 4.3 (0.1)	1.4 (0.6)	1.6 (0.6)
Exposed group (> = 1 visit to a food pantry)	− 0.52 (0.10)	0.11 (0.13)	− 5.4 (2.1)	− 1.8 (0.8)	− 0.95 (0.6)
Difference between exposed and control group	− 0.28 (0.10)	− 0.13 (0.28)	− 1.2	− 3.2 (0.8)	− 2.5 (1.1)
p-value for difference	0.007	0.653	0.606	< 0.001	0.028
Change in outcome per visit	− 0.03 (0.01)	0.06 (0.06)	0.05	− 0.16 (0.04)	− 0.11 (0.04)
p-value	0.005	0.335	0.884	< 0.001	0.007
Change in outcome by intensity of exposure (visits per month)	− 0.12 (0.04)	0.11 (0.11)	1.8 (0.7)	− 1.45 (0.6)	− 0.43 (0.47)
p-value	0.009	0.397	0.018	0.016	0.356

All models in Table 3 adjust for length of time between clinic visits, and an HCP-level random effect. *P*-values are based on robust standard errors

In sensitivity analyses (data not shown), we further explored the association of food pantry usage with HbA1c alone. We found that baseline HbA1c was however strongly and positively associated with the magnitude of decline. However, adjusting for baseline HbA1c levels, or stratifying by baseline HbA1c did not substantially alter the association of number of visits with change in HbA1c. Using logistic regression models, we examined the effects of exposure on the odds of any decline in HbA1c, and the odds of a clinically meaningful decline in HbA1c (i.e., >  − 0.50 percent point decline). The odds of any decline in HbA1c showed a linear association with number of visits (OR = 1.06 per visit, *p* < 0.001), as did the odds of a clinically meaningful decline in HbA1c (OR = 1.04 per visit, *p* = 0.007). When the sample was limited to patients that visited the pantry, the magnitude of HbA1c change with number of visits was attenuated to non-significant; however, the odds of any decline in HbA1c, and the odds of a clinically meaningful decline in HbA1c continued to show a statistically significant association with number of visits.

**Fig. 1:**
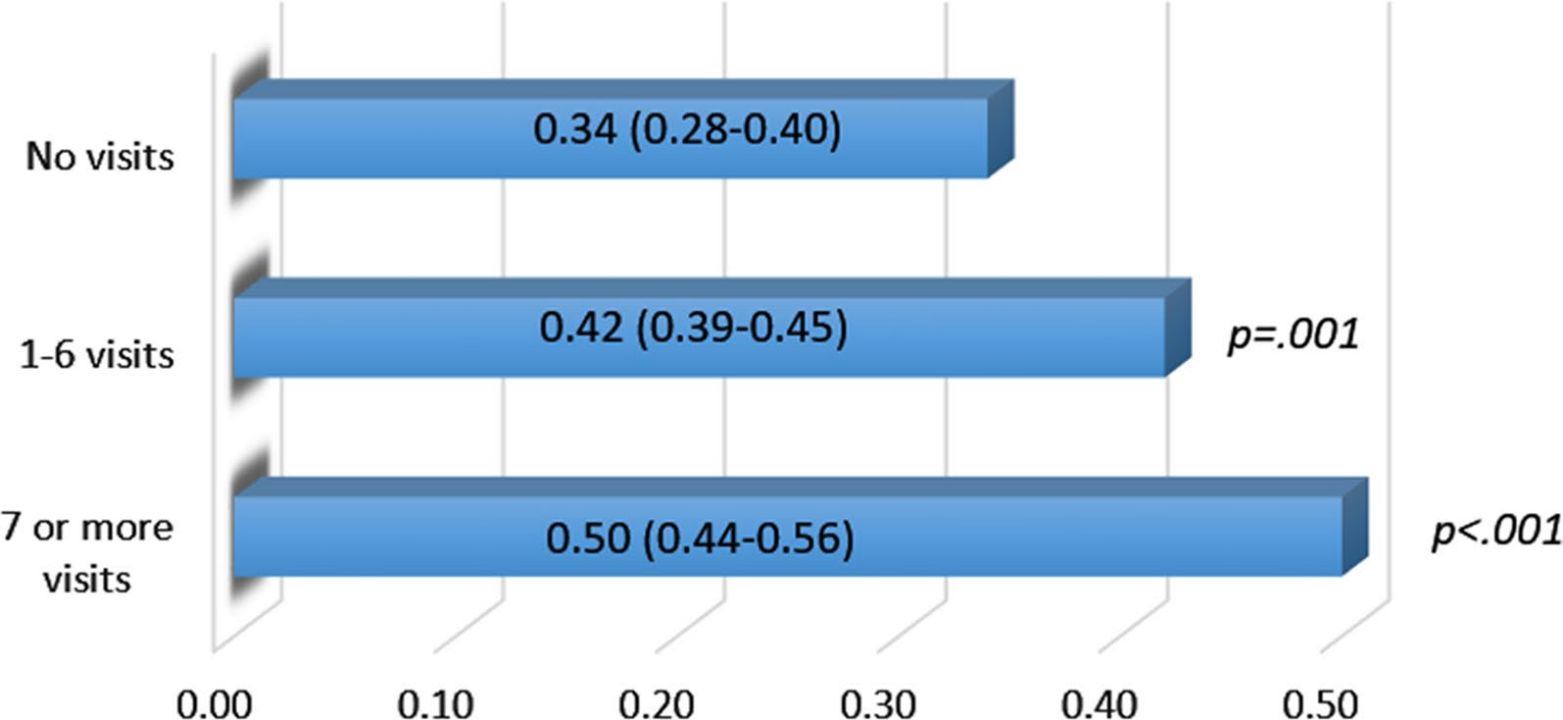
Probability of  > 0.5% decline in A1c over 6 months, by number of visits. P-values shown in Fig. 1 reflect the difference in probability of clinically significant improvements in HbA1c across the indicated dose level, and the no visit dose level

We examined if there was a dose-response association of number of visits (0 visits, 1–6 visits, and 7 or more visits) with the probability of a clinically significant decline in HbA1c (defined as > 0.50 percentage points) over a six-month period [18]. The cut point of 6 was chosen as it is the average number of visits among the exposed. The category ‘7 or more visits’ includes participants with up to 23 visits; they are pooled into a single category here because of small numbers. The average number of visits in the 1–6 visits category is 2.6, while the average number of visits in the 7 or more visits category is 13.7. Figure 1 confirms a clear dose response effect of the number of visits on the probability of a clinically significant decline in HbA1c. At the highest level of ‘dosage’, half of exposed patients experienced a clinically significant decline in HbA1c. It is worth noting that even in the ‘unexposed’ group, a third of the patients experienced a clinically significant decline, likely due to clinical treatment offered at the healthcare partners. Thus, the 50% probability estimate for the highest dose of visits should be interpreted as representing the effect of Food Rx + standard care, and the net effect of Food Rx redemption at that level is closer to a 16% improvement in the probability of clinically significant decline (50–34%) in HbA1c.

## Discussion

This impact analysis of a large redemption-based Food Rx program involving multiple healthcare agencies, and multiple food distribution points provides robust evidence of a modest decline in hemoglobin HbA1c levels associated with redemption of the food prescription. HbA1c levels declined by −0.52, a clinically meaningful decline, over a 6-month period among those patients that redeemed their food prescriptions at least once (for an average of 6 visits), compared to a −0.24 decline among those that chose not to participate, for a net −0.28 percent point change associated with prescription redemption. Apart from favorable HbA1c changes, we did not find substantial changes in BMI, lipids, or blood pressure during the 6-month period of the study.

There are several reasons why these results are credible. First, this magnitude of pre-post change is well within the range of HbA1c improvements reported in a number of food prescription studies. In the most directly comparable study to ours [19], prepacked boxes of diabetes-appropriate foods were distributed once or twice monthly through food pantries to 687 enrolled clients with diabetes across three states over six months. The average pre-post change in HbA1c was −0.15 percent points across all participants, and -0.48 percent points among participants with elevated HbA1c at baseline (> 7.5 percent). In a recent meta-analysis of Food Rx studies [16], the average change in HbA1c across the five included studies was − 0.81 percent points; however, those results were dominated by smaller studies, which are qualitatively different from ours. Second, we found moderate evidence that the magnitude of HbA1c improvements increased with the number of visits to a food pantry, although this evidence was metric-dependent. Third, given that these patients were enrolled through healthcare clinics, a decline in HbA1c levels even among those patients that did not visit a food pantry is only to be expected. Finally, the lack of change in lipids and blood pressure over a six month period that we observed is consistent with the literature [16]. While there is some evidence that food Rx programs can reduce BMI [16], the evidence is based on a limited number of studies, and observed BMI reductions were modest.

Approximately half of patients enrolled by healthcare partners in the program chose not to redeem their prescriptions. This raises the possibility that participants who redeemed prescriptions were selected for characteristics associated with better compliance with medication, for example. Although we had limited covariate information to create a propensity-matched control group from these patients, survey data obtained from a subset of healthcare partners enrollees showed few socioeconomic differences across participants and non-participants. Hence, for the purpose of this analysis, self-selection into exposure (i.e., redeeming prescription at a pantry) was deemed unlikely to result in bias.

The average number of food pantry visits among those that did redeem their vouchers was just six visits, a level that would not be considered a sufficient dose to effect clinically significant changes in HbA1c levels. Although there is some evidence that substantial HbA1c improvements can occur with as few as four redemptions [20], a more plausible explanation is that visits to a food pantry/other source of nutritious foods trigger other exposures or health-protective behaviors that affect HbA1c levels. For example, in the HFB program, patients received health information from staff at the food pantry. This should be seen as part of the treatment package, not as a selection effect.

Even if we can plausibly rule out several sources of selection biases, this study does suffer from some limitations, that arise as an inevitable consequence of the structure and scale of the program and data sharing procedures. These include considerable incomplete data (patients not returning for follow-up visits), and substantial variability in the period between pre- and post-measures. Additionally, exposure status was ascertained by matching unique participant IDs to those presented at food pantries. Given the variety of data sources and the size of the visit level data, in conjunction with limited data handling capacity at some of the smaller healthcare partners data centers, it is highly likely that some enrolled participants were not correctly matched to the food bank client tracking system, and accordingly treated as non-participants. Similarly, the number of visits per participant may be subject to data entry errors. These non-systematic errors in exposure measurement, however, should bias estimates towards the null, and do not undermine our main conclusion. Finally, the lack of socioeconomic covariates limited our ability to conduct subgroup analysis.

## Conclusions

The most important contribution of this study is that it confirms that an effect size of this magnitude can be attained in the setting of a large multi-institution setting for a Food Rx program involving food pantries and healthcare clinics. We are aware of only one other study conducted at this scale [18].There is increasing interest from both philanthropic organizations and healthcare payers in scaling up Food Rx programs as an alternative or supplementary strategy to reduce health disparities and the risk of diabetes, diabetic complications and other related chronic diseases. This model is substantially less costly than a food prescription program that delivers food to patients’ homes, but, as our data shows, has a high risk of insufficient compliance and dose. Although there are scaling and program implementation challenges, they are not insurmountable. Further work in this area should address issues of low motivation of patients to utilize food pantries, which are well-positioned to serve as an important backbone of disease prevention and management for the poor in the United States.

## Limitations

As a quasi-experimental study that lacks a true randomized control group, this study potentially suffers from selection biases, with systematic unmeasured differences between the treatment and control conditions. Although the experimental group and control group were socio-demographically comparable, baseline levels of HbA1c were marginally higher in the exposure group. It is possible that higher levels of HbA1c predispose to better compliance with both Food prescriptions and with standard care medication. To some extent, we controlled for this by examining change scores; addition, we showed that visit frequency was not determined by baseline HbA1c. Another limitation is that we do not have information on actual consumption of healthy food, which presumably mediates improvements in HbA1c. Finally, both the limited sample size for each outcome, and the amount of available socioeconomic information, precluded more detailed analysis that would have allowed investigation of redemption behavior. Despite these limitations, the consistency of the main finding of HbA1c declines across different analyses suggests that these results are robust and credible.

## Data Availability

The results reported here are from a secondary data analysis conducted by researchers at the University of Texas Health Science Center (UTHealth) School of Public Health, with data provided from by the Houston Food Bank under a data use agreement. The data that support the findings of this study are available from the Houston Food Bank but restrictions apply to the availability of these data, which were used under license for the current study, and so are not publicly available. Data are however available from the authors upon reasonable request assuming that the Houston Food Bank grants permission for sharing.

## Footnotes

1 Exposure prevalence varies by patient pool (all enrolled versus those with complete data), as well as by outcome. The percent of patients with complete HbA1c data that were exposed may be different from the percent of patients with complete BMI data.
